# Three-dimensional illusion thermal device for location camouflage

**DOI:** 10.1038/s41598-017-07902-5

**Published:** 2017-08-08

**Authors:** Jing Wang, Yanqiang Bi, Quanwen Hou

**Affiliations:** 1 Beijing Institute of Spacecraft Environment Engineering, Beijing, 100094 China; 2 Smart Materials Laboratory, Department of Applied Physics, Northwestern Polytechnical University, Xi’an, 710129 China

## Abstract

Thermal metamaterials, proposed in recent years, provide a new method to manipulate the energy flux in heat transfer, and result in many novel thermal devices. In this paper, an illusion thermal device for location camouflage in 3-dimensional heat conduction regime is proposed based on the transformation thermodynamics. The heat source covered by the device produces a fake signal outside the device, which makes the source look like appearing at another position away from its real position. The parameters required by the device are deduced and the method is validated by simulations. The possible scheme to obtain the thermal conductivities required in the device by composing natural materials is supplied, and the influence of some problems in practical fabrication process of the device on the effect of the camouflage is also discussed.

## Introduction

Transformation electromagnetics, as a powerful tool to control electromagnetic energy proposed by Leonhardt^1^ and Pendry *et al*.^2^ ten years before, has been studied intensively. Recently, the similar theory extents to heat transfer regime, leading to the theory of transformation thermodynamics^3, 4^, and guides designs of various thermal devices^4–23^.

Due to the possible applications in military and engineering, illusion thermal devices have attracted much attentions. The most famous illusion thermal devices are the thermal cloaks, which cancel the signatures of the objects covered by the cloaks in regions outside the cloaks and create illusions that the objects do not exist. The 2-dimensional thermal cloaks have been designed and verified by many researchers previously^4, 5, 10, 12, 19^. Then, Han *et al*.^14^ and Xu *et al*.^16^ extended the relative work to 3-dimensions. In addition to the cloaks designed at marcoscale, the thermal cloak is even discussed at microscale recently^24^.

In addition to this kind of thermal device, some other kinds of illusion thermal devices and methods are also proposed. He *et al*.^13^ proposed a method to reshape an arbitrary thermal object into another one in theory. Cheng *et al*.^25^ proposed a method to make a good thermal conductor to be disguised as a poor one. In summary, these devices change the attributes of objects such as shapes or thermal properties.

Hou *et al*.^26^ proposed a thermal device for delocalizing the location of the object in 2-dimentional heat conduction regime, which can be used to camouflage the locations of heat sources such as engines in vehicles or boilers in buildings. In addition, the device can be constructed by only one kind of material with anisotropic thermal conductivity. In practice, however, most heat sources are 3-dimensional. Then a 3-dimensional device design and the construction scheme will be more important, which have not been discussed before.

In this paper, an illusion thermal device for 3-dimensional heat conduction is proposed, which can delocalize a heat source covered by the device such that the temperature profile outside the device appears to be produced by a virtual source at another position. The increased dimension leads to some different characteristics in the device compared with that in 2-dimensional cases. Numerical simulations have been performed to validate the method proposed, and an implementation scheme, by means of artificial composite materials, is suggested for constructing the practical thermal device. The effects of some factors involved in the fabrication process for the device are also discussed.

## Theory

To produce an illusional signal for the position of heat source, transformation thermodynamics^4^ can be applied to a device zone covering the physical position and the illusional position of the heat source. During the coordinate transformation, the illusion zone containing the heat source translates from the illusional position to the physical position, while the outer zone and the boundary of the device zone maintain unchanged. Correspondingly, each point in the device zone in illusional space ***r*** changes to a new point in physical space ***r***′. Based on the transformation thermodynamics, the thermal conductivity in each point in real space can be calculated by

1 $$\lambda \text{'}({\boldsymbol{r}}{\boldsymbol{\text{'}}})=A\lambda ({\boldsymbol{r}}){A}^{T}/\det (A),$$

where *λ* is the thermal conductivity on original position in illusional space. *A* ^*T*^ and det(*A*) are transform and determination of *A*, respectively, and *A* is the Jacobi matrix of the coordinate transformation from the illusional space ***r*** to the physical space ***r***′ *A* = ∂(*x*′,*y*′,*z*′)/∂(*x*,*y*,*z*). When Eq. (1) is satisfied, the heat source in physical space will produce a fake temperature signal in the outer zone, to make it look like appearing on the illusional position.

**Figure 1:**
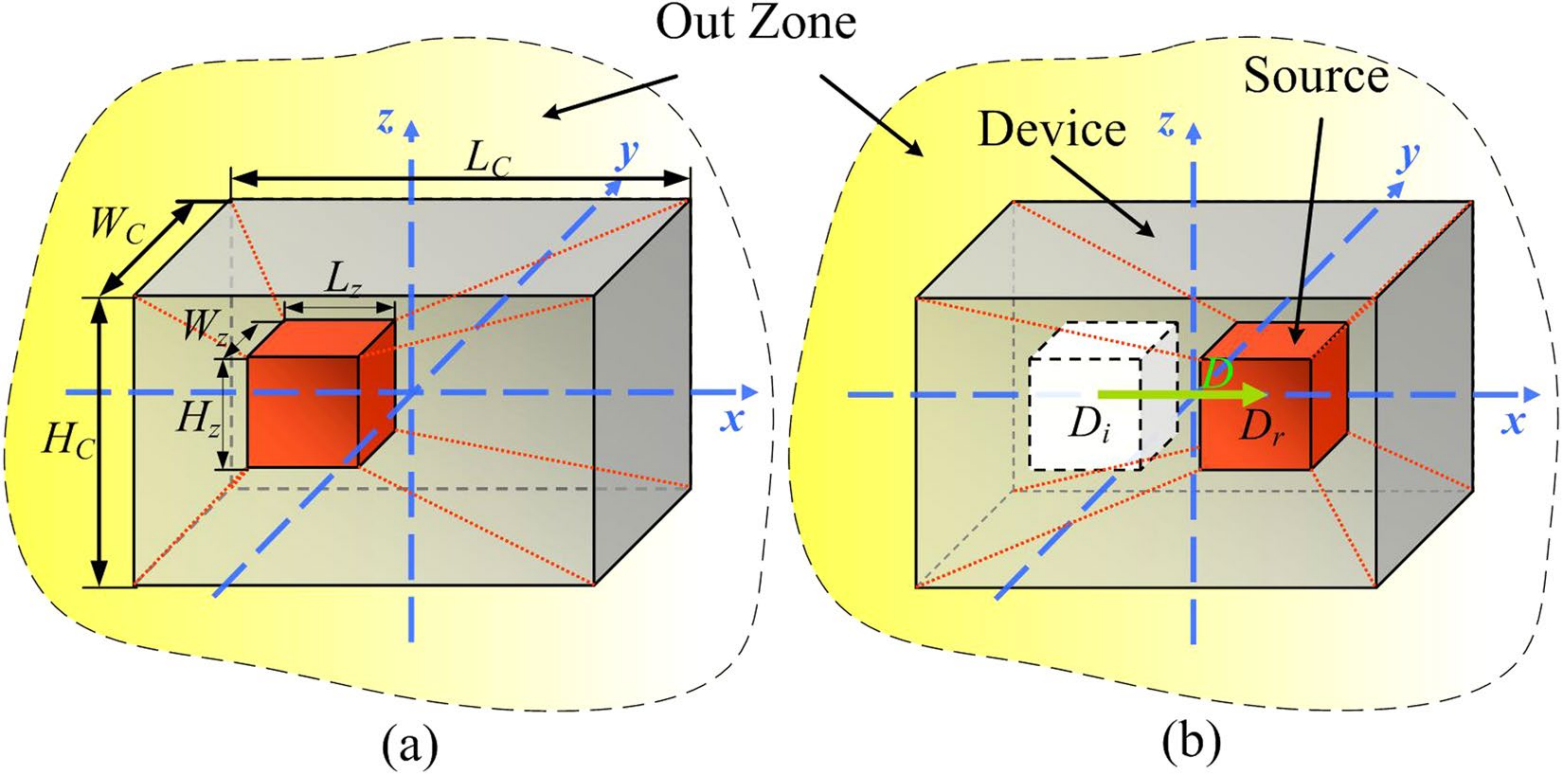
The schematic of the coordinate transformation in the device zone. In the illusional space (**a**), the heater is localized on the left part. In physical space (**b**), the heater is localized on the right. The six pyramids with red dotted lines as bevel edges in the illusional space are then transformed to the six pyramids in physical space correspondingly.

In general, anisotropic and inhomogeneous thermal conductivities are required for realizing the device based on Eq. (1). This increases the fabrication complexity for the device, since many types of materials with different properties will be used. To reduce the types of materials required for constructing the device, a cuboids device with dimension Lc*Wc*Hc (device zone) covering a cuboids illusion zone with dimension Lz*Wz*Hz is designed. The schematic structure is shown in Fig. 1. The corresponding edges of the device zone and the illusion zone are parallel to each other. The coordinate of the center of the illusion zone is (*D*_*i*_, 0, 0) in the illusional space (***r***), and (*D*_*r*_, 0, 0) in physical space (***r***′). To camouflage the position of the heat source embedded in the illusion zone in physical space to the position in illusional space, a coordinate transformation is applied to the device zone, which transforms the inner points of the device in illusional space (Fig. 1(a)) to those in physical space (Fig. 1(b)). The illusion zone is translated along *x* axis during the transformation, and the displacement is defined as *D* = *D*_*r*_ − *D*_*i*_. In both spaces, the center of the device cuboids is set on the original point of the coordinate system.

To simplify the thermal properties of the device, the device zone is divided into 6 pyramids, i.e. *top*, *bottom*, *left*, *right*, *front* and *back* as shown in Fig. 1. The bevel edges of the pyramids are shown as red dotted lines. For each pyramid, a linear coordinate transformation is applied as

2 $$\{\begin{array}{c}x\text{'}={a}_{i}x+{b}_{i}y+{c}_{i}z+{d}_{i}\\ y\text{'}=y\\ z\text{'}=z\end{array},$$

where only *x* coordinate is transformed in different spaces. The coefficients *a*_*i*_, *b*_*i*_, *c*_*i*_ and *d*_*i*_ for the *i*th pyramid are listed in Table 1.

**Table 1:** The coefficients of the coordinate transformation for pyramid zone in the device.

Pyramid	*a*_*i*_	*b*_*i*_	*c*_*i*_	*d*_*i*_
1(*Top*)	1	0	−2*D*/(*H*_*c*_−*H*_*z*_)	*DH*_*c*_/(*H*_*c*_−*H*_*z*_)
2(*Bottom*)	1	0	2*D*/(*H*_*c*_−*H*_*z*_)	*DH*_*c*_/(*H*_*c*_−*H*_*z*_)
3(*Left*)	$\frac{2{D}_{r}+({L}_{c}-{L}_{z})}{2{D}_{i}+({L}_{c}-{L}_{z})}$	0	0	$\frac{D{L}_{c}}{2{D}_{i}+({L}_{c}-{L}_{z})}$
4(*Right*)	$\frac{({L}_{c}-{L}_{z})-2{D}_{r}}{({L}_{c}-{L}_{z})-2{D}_{i}}$	0	0	$\frac{D{L}_{c}}{({L}_{c}-{L}_{z})-2{D}_{i}}$
5(*Front*)	1	2*D*/(*W*_*c*_−*W*_*z*_)	0	*DW*_*c*_/(*W*_*c*_−*W*_*z*_)
6(*Back*)	1	−2*D*/(*W*_*c*_−*W*_*z*_)	0	*DW*_*c*_/(*W*_*c*_−*W*_*z*_)

For simplicity, *D*_*i*_ and *D*_*r*_ are chosen as *D*_*i*_ = −*D*_*r*_ = −*D*/2 in this work. Based on Eq. (1), the thermal conductivities of the *left* and the *right* pyramids are $\lambda ^{\prime} =\lambda \cdot {\rm{diag}}(1+D/{\rm{\Delta }},\frac{1}{1+D/{\rm{\Delta }}},\frac{1}{1+D/{\rm{\Delta }}})$ and $\lambda ^{\prime} =\lambda \cdot {\rm{diag}}(\frac{1}{1+D/{\rm{\Delta }}},$ $1+D/{\rm{\Delta }},1+D/{\rm{\Delta }})$, respectively, where 2Δ = *L*_*c*_ − *L*_*z*_ − *D*. The thermal conductivities *λ*′ of other pyramid zones (*top, bottom, front* and *back*) can also be calculated by Eq. (1). Based on suitable coordinate frames, these thermal conductivities can also be expressed by diagonal tensors *λ*″

3 $$\lambda ^{\prime\prime} =R\lambda ^{\prime} {R}^{T}$$

where *R* is a rotation matrix in 3-dimension. Especially, in the case of *H*_*c*_−*H*_*z*_ = *W*_*c*_−*W*_*z*_ = 2*δ*, the thermal conductivities in these four pyramid zones will have the same value due to the symmetry of the device, and can be expressed as *λ*″ = *λ*⋅diag(*η* ^+^/*η* ^−^,1,*η* ^−^/*η* ^+^), where ${\eta }^{+}={(D/\delta )}^{2}+4+(D/\delta )\sqrt{{(D/\delta )}^{2}+4}$ and ${\eta }^{-}={(D/\delta )}^{2}+4\,-$ $(D/\delta )\sqrt{{(D/\delta )}^{2}+4}$. The rotation matrixes for the four pyramids are listed below.

4 $$\begin{array}{ll}{R}_{1}=[\begin{array}{ccc}\cos \,{\theta }_{1} &  & \sin \,{\theta }_{1}\\  & 1 & \\ -\sin \,{\theta }_{1} &  & \cos \,{\theta }_{1}\end{array}], & {R}_{2}=[\begin{array}{ccc}\cos \,{\theta }_{2} &  & \sin \,{\theta }_{2}\\  & 1 & \\ -\sin \,{\theta }_{2} &  & \cos \,{\theta }_{2}\end{array}]\\ {R}_{5}=[\begin{array}{ccc}\cos \,{\theta }_{5} & \sin \,{\theta }_{5} & \\ -\sin \,{\theta }_{5} & \cos \,{\theta }_{5} & \\  &  & 1\end{array}], & {R}_{6}=[\begin{array}{ccc}\cos \,{\theta }_{6} & \sin \,{\theta }_{6} & \\ -\sin \,{\theta }_{6} & \cos \,{\theta }_{6} & \\  &  & 1\end{array}]\end{array}$$

where

5 $$\begin{array}{l}{\theta }_{1}=\arctan \,(\frac{-{c}_{1}-\sqrt{{{c}_{1}}^{2}+4}}{2}),{\theta }_{2}=\arctan \,(\frac{-{c}_{2}+\sqrt{{{c}_{2}}^{2}+4}}{2})\\ {\theta }_{5}=\arctan \,(\frac{-{b}_{5}+\sqrt{{{b}_{5}}^{2}+4}}{2}),{\theta }_{6}=\arctan \,(\frac{-{b}_{6}-\sqrt{{{b}_{6}}^{2}+4}}{2})\end{array}.$$

Different from 2-dimensional cases, the thermal conductivities required by the device in 3-dimention cannot be the same for all pyramids, no matter how the dimensions of the device is chosen. It means the device cannot be constructed by only one kind of material as in 2-dimensional cases.

## Results

To verify the design proposed above, numerical simulations are implemented. In the simulations, a heat source (*Q* = 500000 W/m^3^) embedded in a background material is camouflaged to another position *D* = 5 cm away. The heat source is a cube with dimension of *L*_*z*_ = *W*_*z*_ = *H*_*z*_ = 5 cm, and the center of the source is on point (2.5 cm, 0, 0). The background material is stainless steel in cuboids with dimension of *L*_*b*_ × *W*_*b*_ × *H*_*b*_ = 24 × 20 × 20 cm^3^. The thermal conductivity of the whole system is *λ* = 30 W/(m⋅K). To achieve the goal of camouflage, the heat source is covered by a device with dimension of *L*_*c*_ × *W*_*c*_ × *H*_*c*_ = 20 × 15 × 15 cm^3^. The required thermal conductivity of each pyramid in the device can be calculated by Eq. (1), i.e.

$$\begin{array}{c}{\lambda }_{1}\text{'}=[\begin{array}{ccc}60 &  & -30\\  & 30 & \\ -30 &  & 30\end{array}][W/({\rm{m}}\cdot {\rm{K}})],{\lambda }_{2}\text{'}=[\begin{array}{ccc}60 &  & 30\\  & 30 & \\ 30 &  & 30\end{array}][W/({\rm{m}}\cdot {\rm{K}})],{\lambda }_{3}\text{'}=[\begin{array}{ccc}60 &  & \\  & 15 & \\  &  & 15\end{array}][W/({\rm{m}}\cdot {\rm{K}})],\\ {\lambda }_{4}\text{'}=[\begin{array}{ccc}15 &  & \\  & 60 & \\  &  & 60\end{array}][W/({\rm{m}}\cdot {\rm{K}})],{\lambda }_{5}\text{'}=[\begin{array}{ccc}60 & 30 & \\ 30 & 30 & \\  &  & 30\end{array}][W/({\rm{m}}\cdot {\rm{K}})],{\lambda }_{6}\text{'}=[\begin{array}{ccc}60 & -30 & \\ -30 & 30 & \\  &  & 30\end{array}][W/({\rm{m}}\cdot {\rm{K}})],\end{array}$$

In suitable reference frames, they can be expressed as diagonal tensors, i.e. *λ*_3_″ = diag(60,15,15)[W/(m⋅K)], *λ*_4_″ = diag(60,60,15)[W/(m⋅K)] and *λ*_1_″ = *λ*_2_″ = *λ*_5_″ = *λ*_6_″ = diag(78.5,30,11.5)[W/(m⋅K)]. The whole system is exposed to air with temperature *T*_*c*_ = 293.15 *K*. The convective heat transfer condition (*h* = 10 W/(m^2^⋅K)) is applied to all outer surfaces of the sample.

**Figure 2:**
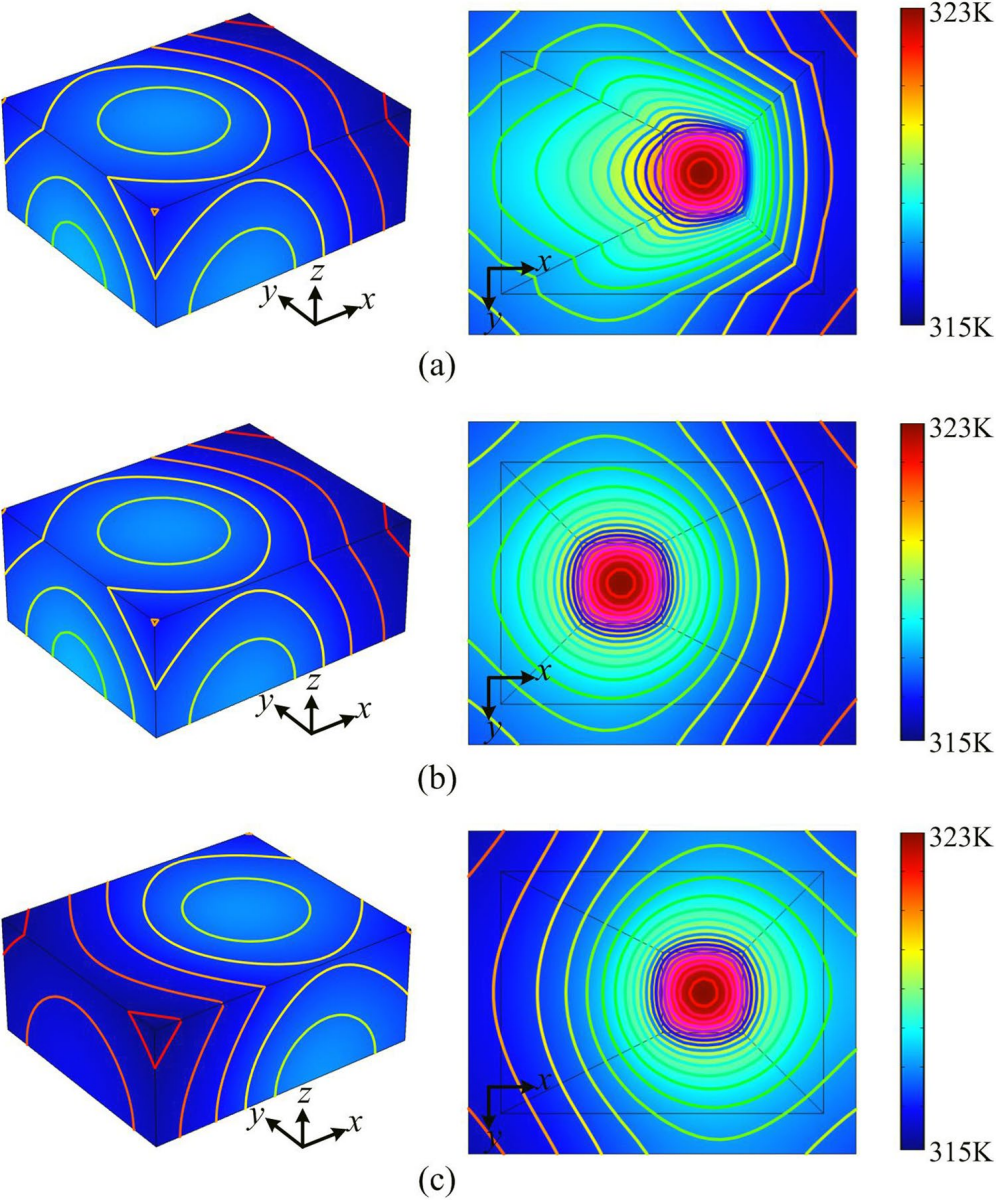
The simulated temperature profiles produced by heat sources. (**a**) The heat source is placed on the right part of the system and covered by the illusion thermal device. (**b**) The heat source is placed on the left part of the system without thermal device. (**c**) The heat source is placed on the right part of the system without thermal device.

The temperature profiles produced by the heat source are shown in Fig. 2(a). Due to the symmetry of the system, only a half part (z > 0) of the system is simulated. For comparison, samples without device are also simulated. The results of samples with the heat source embedded in the left part (center of the source on (−2.5 cm, 0, 0)) and in the right part (center of the source on (2.5 cm, 0, 0)) are shown in Fig. 2(b) and (c), respectively. Usually, when the heat source is placed on the right, the maximum temperature on top surface of the system should appear on the right part of the surface, as shown in Fig. 2(c). From Fig. 2(a), however, we can see that when the device exists, the heat source on the right part produces the same temperature profile on the surface of the system as that produced by the heat source on the left part (Fig. 2(b)), although they have different positions for heat sources. Since the maximum temperature appears on the left part of the top surface, it looks like that the heat source is also on the left part of the sample. In this sense, the device results in the position camouflage effect in heat conduction regime, which may be applied to camouflage the positions of the heat sources in various vehicles or buildings.

## Discussions

**Figure 3:**
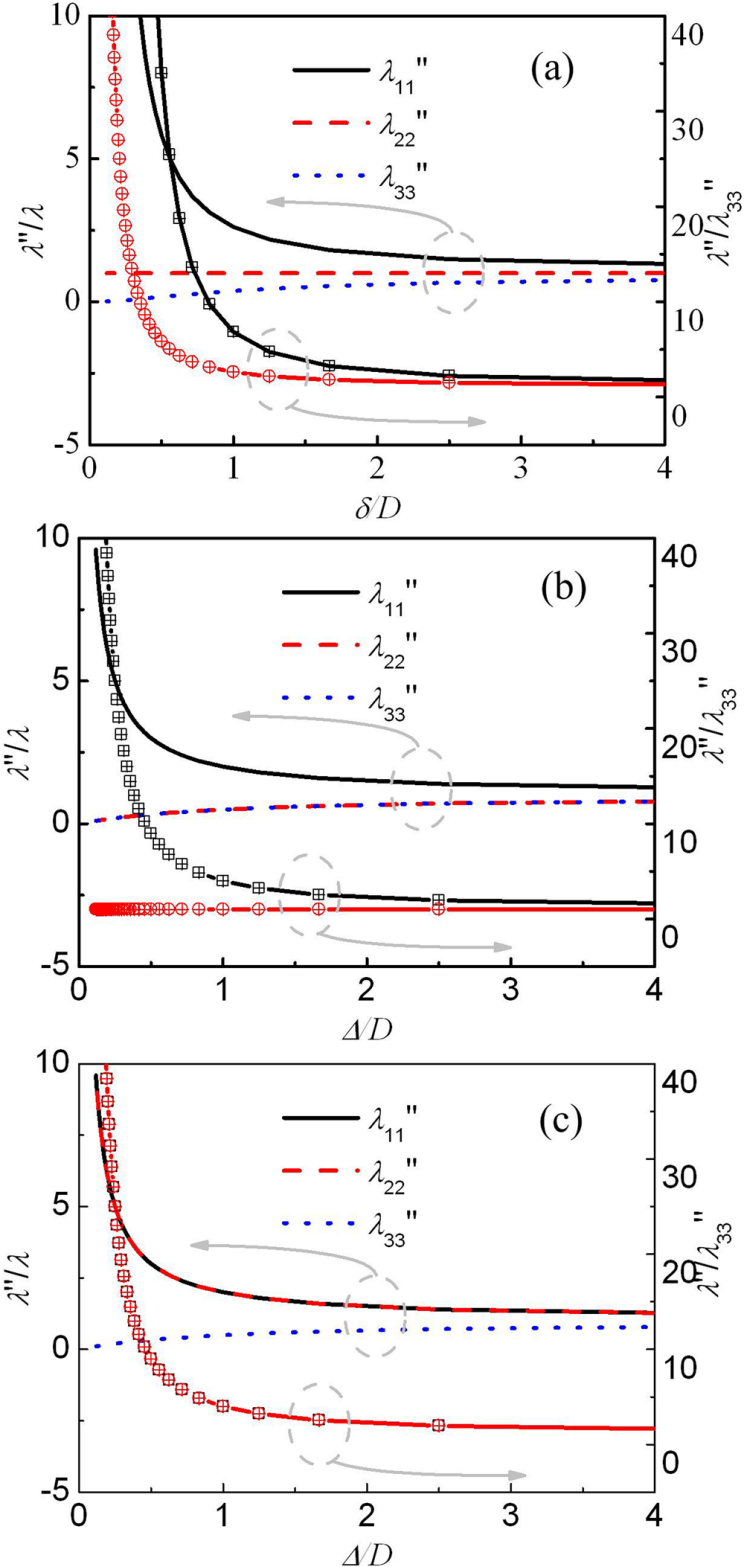
Thermal conductivities and anisotropies of different pyramids varying with the dimensions of the device. (**a**) Properties of the top, bottom, front and back pyramids varying with thickness *δ*. (**b**) Properties of the left pyramid varying with thickness Δ. (**c**) Properties of the right pyramid varying with thickness Δ.

For practical applications, the device with different dimensions can be designed, and will possess different thermal property distributions and different anisotropies. Figure 3 shows the thermal conductivity and anisotropy varying with the dimension of the device. As the device becomes thin (*δ* decreasing), the anisotropy of thermal conductivity (*λ*_11_″/*λ*_33_″,*λ*_22_″/*λ*_33_″) of the four pyramids (*top, bottom, front* and *back*) increases. Same to this tendency, the anisotropies of thermal conductivity of the *left* pyramid and *right* pyramid also increase as the device becomes short (*Δ* decreasing). Especially, when *δ*/*D* < 1 and Δ/*D* < 1, the anisotropy of thermal conductivity increases rapidly as the device scale decreases. It means that the small device requires materials with high anisotropies. In addition, the anisotropy required by each pyramid is different. In the *left* and the *right* pyramid, two components of the thermal conductivity tensors are the same, i.e. *λ*_11_″ > *λ*_22_″ = *λ*_33_″ for the *left* pyramid and *λ*_11_″ = *λ*_22_″ > *λ*_33_″ for the *right* pyramid, while in the other pyramids, thermal conductivity tensor has three different components (*λ*_11_″ > *λ*_22_″ > *λ*_33_″). Due to these characteristics, the device cannot be constructed by only one kind of material with anisotropic thermal conductivity, but three kinds of materials with different properties at least. This is different from the cases in 2-dimensional heat conduction, where only one kind of material with anisotropic thermal conductivity is required to construct the device. Even though, this design is still simpler than some other structures, since in each pyramid, the thermal conductivity required is homogeneous.

**Figure 4:**
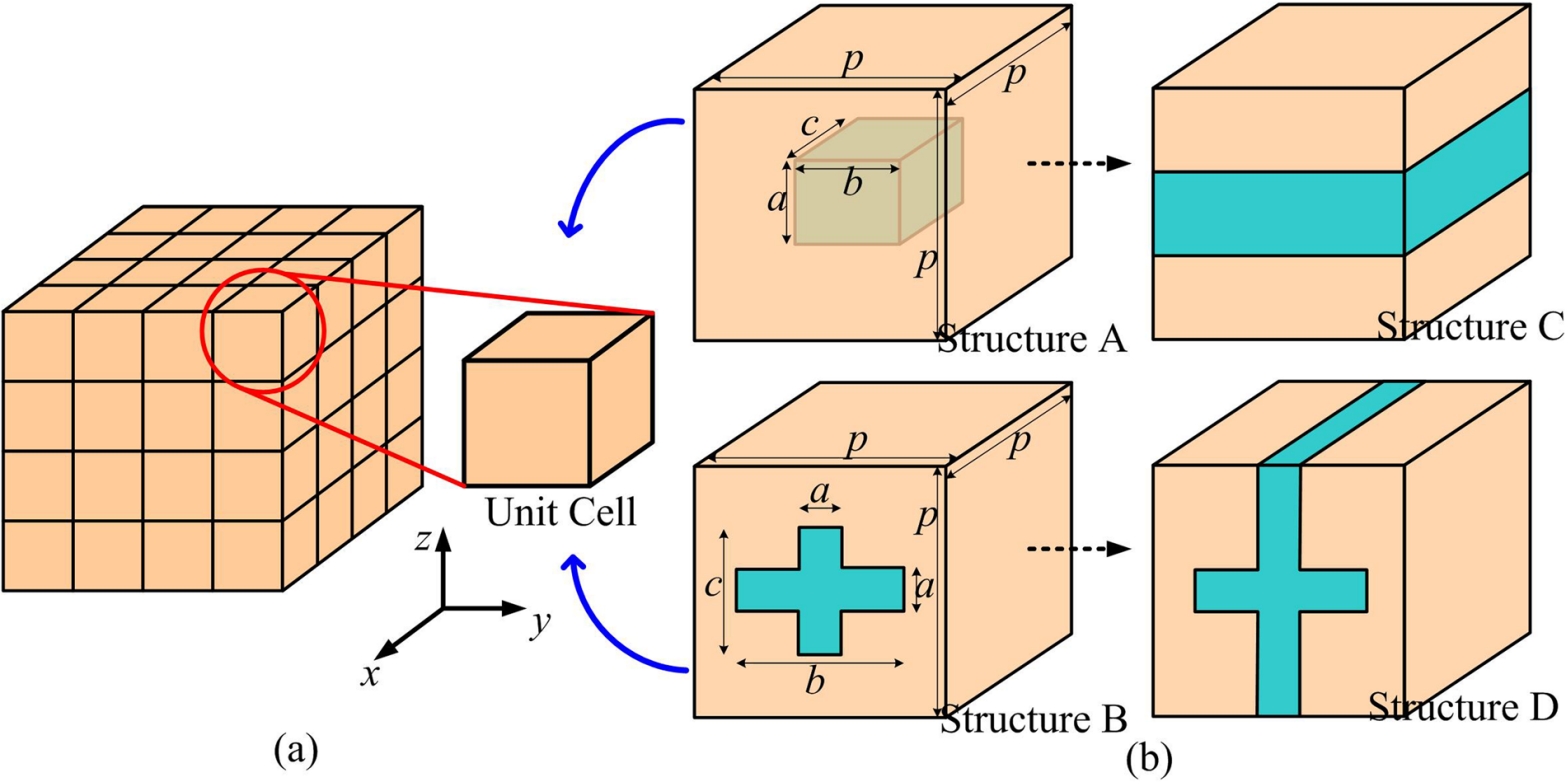
The schematic to construct the artificial materials used in the device. (**a**) The artificial material with required thermal conductivity can be constructed by unit cells in regular arrangement. (**b**) The unit cells with different structures are designed to meet the requirements in different pyramids in device. In structure A, a cubic hole is drilled in the base material (brass). In structure B, a cross-shaped through-hole is drilled on the base material. Structure C and structure D are simplified structures from structure A and B, respectively.

Many natural materials possess anisotropic thermal conductivities, but it is not easy to find appropriate materials satisfying all the requirements of the device exactly. A more convenient method is to construct artificial materials by composites with designed patterns, which has the advantage of tunable thermal conductivity and tunable anisotropy, and has been applied in many other researches^5, 11, 19^. For the device simulated above, the three required thermal conductivities can be achieved by drilling regular patterns (air filled) in brass (98 W/(m⋅K)) as shown in Fig. 4. The whole composites can be regarded as regular arrangements of the cubic units with different structures for different effective thermal conductivities. In structure A, a cubic hole is designed. When *b*/*p* = *c*/*p* = 0.95 and *a*/*p* = 0.4, the effective thermal conductivity of the composite meets the requirement of material used in *right* pyramid (material 1). In structure B, a cross-shaped through-hole is designed. When *a*/*p* = 0.23 and *b*/*p* = *c*/*p* = 0.93, the effective thermal conductivity of the composite meets the requirement of material used in *left* pyramid (material 2). When *a*/*p* = 0.1, *b*/*p* = 0.9 and *c*/*p* = 0.98, structure B realizes another effective thermal conductivity, which meets the requirement of material used in other pyramids (material 3).

**Figure 5:**
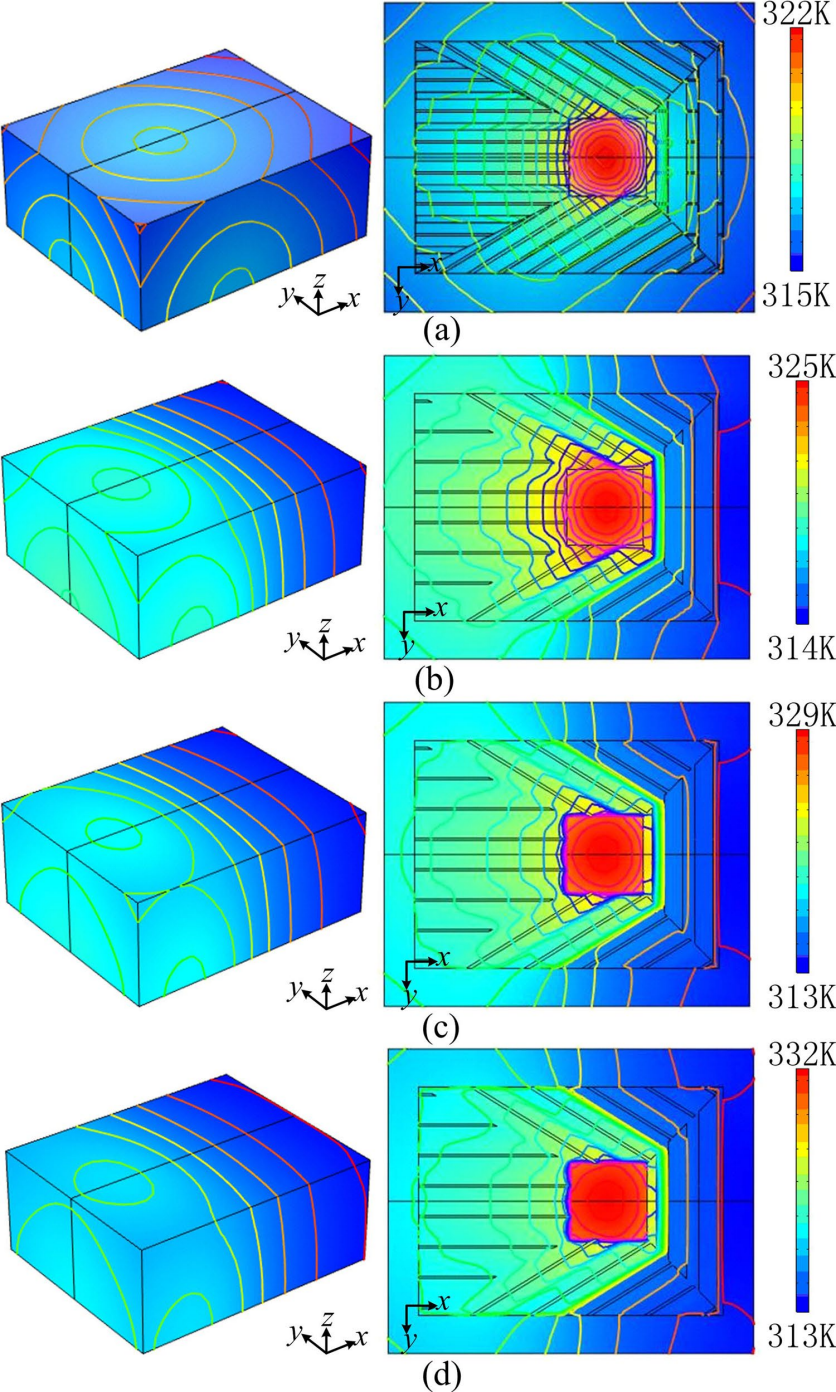
The simulated temperature profiles produced by heat sources for different thermal devices: (**a**) device with designed structures; (**b**) device with simplified structures; (**c**) device with simplified structures (with ITR 0.0005 K⋅m^2^/W); and (**d**) device with simplified structures (with ITR 0.001 K⋅m^2^/W).

**Figure 6:**
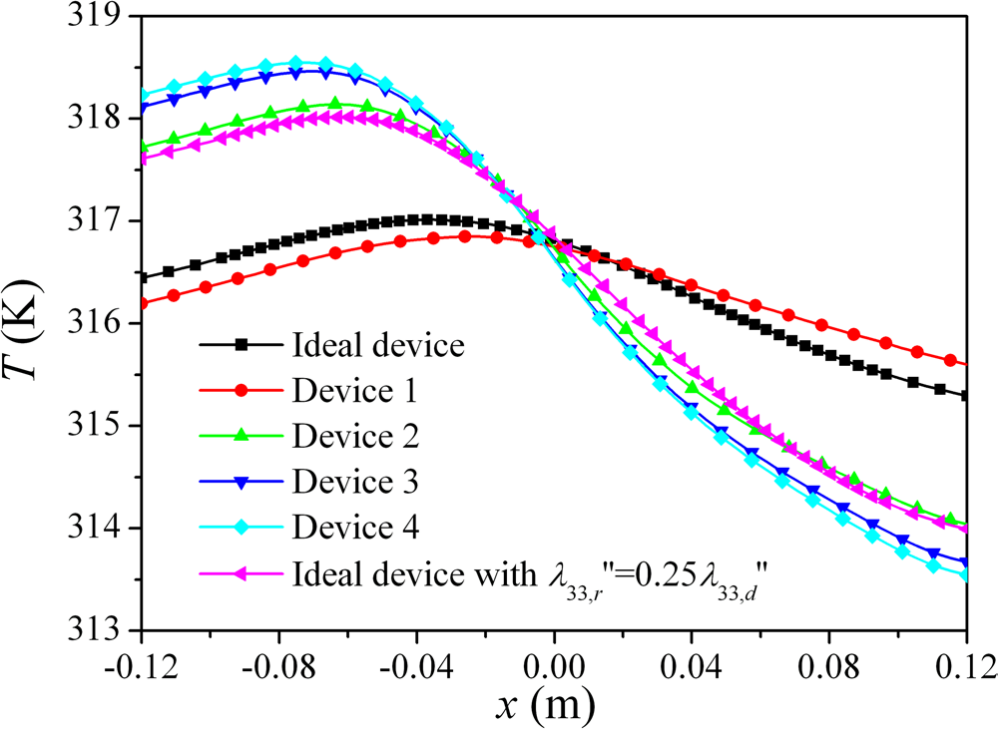
The temperature profile along *x*-axis on the top surface of the system. *λ*_33,*r*_″ and *λ*_33,*d*_″ represent the decreased and the originally designed minimum components of thermal conductivity tensor, respectively. Therefore, to achieve good effect of camouflage, good process accuracy and low ITR values are required in the construction process for the device.

Based on the structures A and B discussed above, a thermal device (Device 1) with the unit length of the structures *p* = 2 cm is designed and simulated. The temperature profile is shown in Fig. 5(a). In this case, although the heat source is on the right part of the system, the highest temperature value on the top surface appears on the left part. Comparing Fig. 5(a) with Fig. 2(a), we can see that the temperature profile produced by Device 1 is nearly the same as that produced by an ideal device in Fig. 2(a), which means that the thermal device with this construction can work as ideal devices proposed theoretically. The temperature distributions along *x* axis on top surface for these two devices are plotted in Fig. 6. The very small distinction between the two results further indicates the feasibility of this structured construction method. This distinction mainly results from the large scale of the unit cell in the design, which makes the effective medium theory valid approximately in this case. If small unit cells are applied, the structured device would be expected to have better camouflage effects.

Although the thermal conductivities required can be realized by designed structures, the fabrication process will be complicated. Especially, the accuracy of structures with small scales may not be guaranteed exactly in the fabrication process. For example, for material 1, the thinnest brass wall in structure A is only 1 mm thick, and for material 3, the thinnest wall in structure B is only 0.4 mm thick. These thicknesses make the wall difficult to be fabricated and cracked easily. Hence, simplified structures can be considered as replacements. For material 1, we change *b/p* = *c/p* = 0.95 in structure A to *b/p* = *c/p* = 1, which leads to the structure C in Fig. 4(b). For material 3, *c/p* = 0.98 in structure B is changed to *c/p* = 1, which leads to the structure D in Fig. 4(b). Plastic foams (*λ* = 0.04 W/(m⋅K)) can be used to fill in the vacancy of the simplified structures to provide mechanical supports for the device. The temperature profile simulated based on this simplified device (Device 2) is shown in Fig. 5(b). This device still camouflages the right heat source to the left part of the system. Compared with the ideal device, however, this device results in a large departure in temperature profile. In the simplified device, the neglecting of the thin walls in the unit cells leads to the decrease of the components of thermal conductivity tensors along corresponding directions, and then alters the temperature profile. This can be verified by an additional simulation. In this simulation, an ideal device but with decreased minimum components of thermal conductivity tensors (only 25% of original designed values) in each pyramid is applied. The temperature distribution along *x* axis on top surface for this case is plotted in Fig. 6, which almost coincides with the temperature distribution produced by Device 2.

The interface thermal resistance (ITR) between different parts or different materials is another factor of importance affecting the camouflage effect of the device. To investigate the effect of ITR, we simulated the heat conduction based on simplified device with two different ITR values between different materials, i.e. 0.0005 K⋅m^2^/W(Device 3) and 0.001 K⋅m^2^/W(Device 4). The temperature profiles are shown in Fig. 5(c) and (d), and the temperature distributions along *x* axis on top surface are plotted in Fig. 6. Compared with the case with no ITR (Device 2), the ITRs further alter the temperature profiles, and lead to more left position of the highest temperature on top surface and higher value of the temperature on top surface. Higher ITR values will result in larger alterations in temperature profiles. All these phenomena stem from the decrease of effective thermal conductivities caused by ITRs.

## Conclusions

In summary, an illusion thermal device for 3-dimensional stationary heat conduction has been designed based on the transformation thermodynamics. This device can delocalize a heat source covered by it such that the temperature profile outside the device appears to be produced by a virtual source at another position. The proposed method has been validated by simulations on a designed system consisted of stainless steel as background material, and it can be also used to other cases. The effect of the device dimension on the thermal conductivity and anisotropy required in the device is discussed. Some structures are proposed as the unit cells of the artificial materials to meet the requirement for the thermal conductivities in the device. In addition, the possible effects of the simplified structures and the interface thermal resistance in fabrication process are discussed by simulations.

### Data availability statement

All data generated or analysed during this study are included in this published article.
